# The effect of maternal alcohol and drug abuse on first trimester screening analytes: a retrospective cohort study

**DOI:** 10.1186/s12884-020-03171-9

**Published:** 2020-09-25

**Authors:** Anni Lehikoinen, Raimo Voutilainen, Jarkko Romppanen, Seppo Heinonen

**Affiliations:** 1 Department of Pediatrics, Kuopio University Hospital, P.O. Box 100, 70029 Kuopio, Finland; 2 Department of Pediatrics, University of Eastern Finland, P.O. Box 1627, FI-70211 Kuopio, Finland; 3 Eastern Finland Laboratory Centre Joint Authority Enterprise (ISLAB), P.O. Box 1700, 70211 Kuopio, Finland; 4 Department of Obstetrics and Gynecology, Helsinki University Hospital, P.O. Box 140, 00029 Helsinki, Finland; 5 Department of Obsteterics and Gynecology, University of Helsinki, P.O. Box 63, 00014 Helsinki, Finland; 6 Department of Obsteterics and Gynecology, Kuopio University Hospital, P.O. Box 100, 70029 Kuopio, Finland

**Keywords:** First trimester screening, PAPP-A, free β-hCG, nuchal translucency, alcohol, smoking, small for gestational age

## Abstract

**Background:**

The purpose of this study was to determine whether first trimester trisomy screening (FTS) parameters are affected by alcohol and drug use.

**Methods:**

A routine combined FTS including measurements of maternal serum levels of free β-human chorionic gonadotropin subunit (free β-hCG) and pregnancy-associated plasma protein A (PAPP-A) were measured at 9–11 weeks of gestation, and fetal nuchal translucency thickness (NTT) at 11–13 weeks of gestation. In total 544 women with singleton pregnancies [71 alcohol and drug abusers, 88 smokers, 168 non-smokers delivering a small for gestational age (SGA) child, and 217 unexposed control women] were assessed.

**Results:**

Free β-hCG levels were higher in alcohol and drug abusing than in unexposed pregnant women [mean 1.5 vs. 1.2 multiples of medians (MoM); *P* = 0.013]. However, stepwise multiple linear regression analyses suggested that smoking could explain increased free β-hCG. Additionally, we observed lower PAPP-A levels in the smoking mothers (0.9 vs. 1.2 MoM; *P* = 0.045) and in those giving birth to an SGA child compared to the controls (1.1 vs.. 1.2 MoM; *P* < 0.001). Fetal NTT did not differ significantly between any of the groups.

**Conclusions:**

The present study shows increased free β-hCG levels in alcohol and drug abusers, but maternal smoking may explain the result. Maternal serum PAPP-A levels were lower in smoking than non-smoking mothers, and in mothers delivering an SGA child. However, FTS parameters (PAPP-A, free β-hCG and NTT) seem not to be applicable for the use as alcohol biomarkers because of their clear overlap between alcohol abusers and healthy controls.

## Synopsis

Higher free β-hCG levels and unaltered PAPP-A levels were found in alcohol-abusing pregnant mothers. Prevalent smoking among the alcohol and drug abusing mothers may explain partly the higher free β-hCG levels.

## Background

One of the challenges for accurate detection and timely treatment of children with fetal alcohol exposure is the difficulty to confirm alcohol exposure during pregnancy, because we lack a reliable biomarker of alcohol abuse [1–3]. The use of available biomarkers of alcohol consumption are hampered by a number of problems: the time window for detection of alcohol use is not sufficient, the biomarker may be insensitive or unspecific, the use of the biomarker has not been validated, or pregnancy itself affects the behaviour of the biomarker [4, 5]. A combination of several biomarkers increases accuracy, but proper validation of such combinations has not been performed in pregnant women [1, 6, 7].

A combined first trimester screening (FTS) test for chromosomal abnormalities includes measurements of pregnancy-associated plasma protein A (PAPP-A) and free β-human chorionic gonadotropin subunit (free β-hCG) from maternal serum, fetal nuchal translucency thickness (NTT), and recording the mother´s age. This combination identifies 85–95% of all fetuses with trisomies 21, 18 and 13, at a false positive rate of 5% [8].

PAPP-A and free β-hCG are known to be influenced by maternal and pregnancy variables such as gestational age, maternal weight, smoking and ethnic background [9]. There is growing evidence that decreased PAPP-A is associated with a delivery of a small for gestational age (SGA) child at the end of pregnancy, even though the systematic review and meta-analysis of Morris et al. [10] showed that the sensitivity of PAPP-A to predict the birth of an SGA child remains low. Previous experiments with human placental cell lines and extraction analyses using human placental samples demonstrated that ethanol exposure increased hCG production. Thus, hCG was suggested for a candidate surrogate biomarker of prenatal ethanol exposure [11]. The influence of drug abuse on first trimester screening parameters has not been previously reported. However, it seems that maternal opioid use does not significantly affect second trimester free β-hCG levels [12].

To our knowledge, the influence of alcohol use on the first trimester screening parameters has not previously been reported. The aim of this study was to determine whether alcohol abuse has effects on the first trimester trisomy screening parameters (NTT, PAPP-A and particularly on free β-hCG) and whether any of these could be used as a biomarker of alcohol use during early pregnancy.

## Methods

**Fig. 1:**
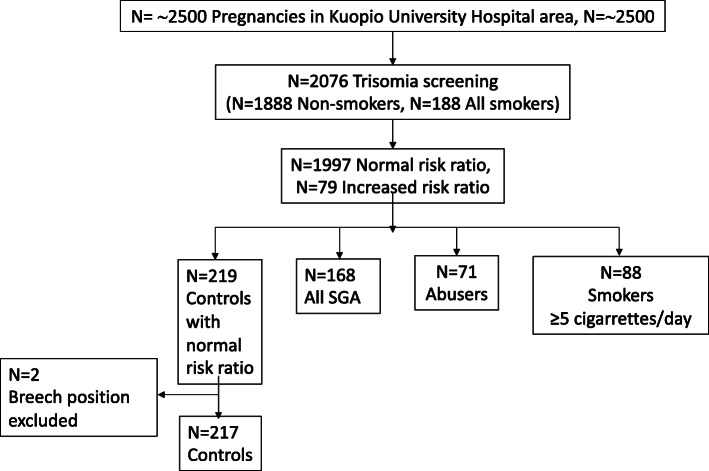
Flowchart showing the number of included and excluded subjects. The discrepancy in the number of the smokers in different boxes is explained by the definition of the final Smokers group (≥ 5 cigarettes/day). Control mothers having increased risk ratio were excluded. Every 8th mother was selected randomly out of all the criteria fulfilling non-smoking mothers to obtain an appropriate number of controls. Thirty-three (42.3%) out of the 71 Abusers were smoking (≥ 5 cigarettes/day) during the pregnancy

This is a retrospective cohort study of pregnant women participating in routine combined FTS for trisomy 21 in the Kuopio University Hospital area in Central Finland. The pregnancies, recorded during FTS in routine maternal care between June 2010 and June 2011, were searched from the medical database. The pregnancy and birth outcomes were evaluated, and 544 study samples were selected out of all pregnancies during that time in the Kuopio University Hospital region (approximately 2500 pregnancies in total) (Fig. 1). A risk ratio < 1:250 was considered normal. The mothers having increased risk ratios (including trisomies, fetal abnormalities, vanishing twins) were excluded from the control group to keep it as “normal” as possible for revealing the possible effect of alcohol and drug exposure on screening parameters.

The final study group included 71 alcohol and possibly drug abusing mothers (“Abusers”), and for comparison all 88 Smokers (five or more cigarettes per day during the pregnancy) having no history of alcohol or drug abuse and all 168 non-smoking (not alcohol or drug abusing) mothers having later given birth to an SGA child (defined here as birth weight below the 10th percentile for gestational age and) (“SGA mothers”). These comparison groups were included, since giving birth to an SGA baby and smoking are common among alcohol abusing mothers. We compared the study groups with 217 non-smoking control mothers having later delivered a normal-sized newborn (birth weight between the 10th and 90th percentile) (“Controls”). The inclusion criteria for the Controls were singleton pregnancy, non-complicated vaginal birth (cephalic presentation), and normal outcome: the mother or the newborn did not require pre- or postnatal follow-up, care or interventions more than considered to be routine. The Controls were healthy women who did not have any other diagnosis at the time of the delivery than a spontaneous, normal parturition according to ICD-10 criteria. Every 8th mother was selected randomly out of all the criteria fulfilling non-smoking mothers to obtain an appropriate number of controls. All mothers were Caucasians by their ethnic background.

The alcohol and drug using pregnant women followed in the maternity clinic of the Kuopio University Hospital had been referred by general practitioners due to concerns aroused by alcohol or drug abuse. The alcohol Use Disorder Identification Test (AUDIT) [4, 5] was used to identify mothers with harmful patterns of alcohol consumption. The AUDIT questionnaire is a validated test used to determine if a person is at risk for alcohol abuse problems. The inclusion criteria for the Abusers were a total AUDIT score of eight or more, alcohol use during the ongoing pregnancy or any alcohol/drug abuse before or during the ongoing pregnancy. The previous risk users were defined as mothers having any previous use of IV drugs, rehabilitation due to drug abuse or long-term use of opioids, stimulants or other drug abuse. Mothers who were Hepatitis C virus antibody positive, in drug or alcohol substitute treatment or committed themselves to drug and/or alcohol abstinence, and mothers who had quit all drug abuse when noting to be pregnant were also defined as previous risk users. After data and outcome collection we did not follow up the mothers or the children.

FTS was performed according to the recommendations of the Finnish ministry of Social Affairs and Health [13]. The screening parameters included PAPP-A and free β-hCG analyses from maternal serum, and fetal NTT measurements. Serum samples for PAPP-A and free β-hCG measurements were collected in maternity care units between gestational weeks 9 + 0 and 11 + 6. Blood samples were allowed to clot at room temperature for 30 min, centrifuged, separated, and stored at + 4 °C. Serum samples were delivered to the Eastern Finland Laboratory Centre in Kuopio refrigerated or frozen and stored at -20 °C. Serum concentrations of free β-hCG and PAPP-A were analyzed by time-resolved fluoroimmunoassay according to the manufacturer’s instructions (PerkinElmer Life and Analytical Sciences, Wallac, Turku, Finland) and the routine first trimester aneuploidy screening protocol. The intra- and interassay coefficients of variation (CV) were < 1.8% and < 3.7% for PAPP-A, and < 2.3% and < 4.1% for free β-hCG, respectively. The CVs were determined in 20 aliquots of two serum pools analyzed in either the same or consecutive runs. The calibrators covered the ranges 10-2000 mU/L for PAPP-A and 2-200 ng/ml for free β-hCG. Serum samples were diluted 5-fold prior to the assay of PAPP-A.

The fetal NTT measurements were performed at healthcare centres or the Kuopio University Hospital Clinic by ultrasound-trained mid-wives and gynecologists between the gestational weeks 11 + 0 and 13 + 6. The crown rump length of the fetus obtained from the ultrasonography examination determined the gestational age. The concentrations of the serum markers and the fetal NTT measures were converted to multiples of medians (MoM). The total trisomy risk for trisomy 21, free β-hCG MoM, PAPP-A MoM and NTT MoM were calculated using LifeCycle software version 2.2 (PerkinElmer Life and Analytical Sciences). The correction factors for free β-hCG were maternal weight, diabetes and smoking status, and for PAPP-A maternal weight and diabetes status. At the time of the analysis the smoking correction factor for free β-hCG was 0.82.

This study was approved by the Research Ethics Committee of Kuopio University Hospital. All study participants provided an informed written consent.

### Statistical analyses

Data management and the statistical analyses were performed using SPSS 19 and 21 (SPSS Inc., Chicago, IL, USA). The continuous variables were tested with the independent samples t-test, if normally distributed. The study groups were compared with the Controls. PAPP-A, free β-hCG and NT showed normal distribution. The total trisomy risk showed normal distribution after log10-transformation. The Chi-square test was used to analyze dichotomous variables. If there were fewer than five units in any of the classes, the Fischer´s exact test was used.

Stepwise multiple linear regression analysis was applied to explain free β-hCG and PAPP-A variation. *P*-value less than 0.05 was considered significant. The following factors were included in the analyses: confirmed maternal alcohol use, smoking, drug abuse, and giving birth to an SGA baby. In addition, a combination of (1) maternal alcohol use and smoking, and (2) maternal alcohol use and giving birth to an SGA baby were included in the regression analyses. The results of the regression analyses were shown as beta coefficients (B) and standardized coefficients (beta).

## Results

The main characteristics of the pregnancies, deliveries and newborns are depicted in Tables 1 and 2. The mean AUDIT score among the Abusers was 15.3 (SD 9.0) and smoking was highly prevalent (42.3%) in this group. The Abusers and Smokers were slightly younger, and the SGA mothers older than the Controls. The Smokers were somewhat heavier than the Controls prior to and at the beginning of the pregnancy. The SGA mothers and Abusers were more often nulliparous than the Controls. The SGA mothers were shorter and had more often arterial hypertension and preeclampsia than the Controls (Table 1).

**Table 1:** Maternal and pregnancy characteristics of the study groups

	Controls (*n* = 217)	Abusers (*n* = 71)	*P*	Smokers (*n *= 88)	*P*	SGA mothers (*n *= 168)	*P*
Maternal age (years,mean ± SD)	29.1 ± 4.5	25.6 ± 6.1	< 0.001	27.1 ± 5.8	0.004	30.2 ± 5.3	0.028
Maternal height (cm, mean ± SD)	165.4 ± 5.4	165.2 ± 4.7	0.803	164.1 ± 5.8	0.053	163.4 ± 5.8	0.001
Weight at the beginning of pregnancy (kg, mean ± SD)	64.2 ± 11.4	66.7 ± 14.0	0.140	70.7 ± 16.2	0.001	66.4 ± 37.5	0.423
BMI prior to pregnancy (kg/m^2^, mean ± SD)	22.9 ± 4.0	23.9 ± 4.9	0.150	25.8 ± 5.9	< 0.001	24.5 ± 15.4	0.162
Weight gain during pregnancy (kg, mean ± SD)	12.8 ± 4.2	14.0 ± 6.3	0.217	13.3 ± 6.9	0.610	11.7 ± 4.4	0.03
Nulliparous, n (%)	60 (27.6)	49 (69.0)	< 0.001	29 (33.0)	0.356	97 (57.7)	< 0.001
Arterial hypertension, n (%)	16 (7.4)	7 (9.9)	0.376	10 (11.4)	0.175	34 (20.2)	< 0.001
Preeclampsia, n (%)	0 (0)	2 (2.8)	NA	6 (6.8)	NA	11 (6.5)	NA
Alcohol use before pregnancy, n (%)	90 (41.5)	38 (53.5)	0.081	41 (46.6)	0.432	76 (45.2)	0.452
Smoking during pregnancy (≥ 5 cigarrettes/day), n (%)	0 (0)	30 (42.3)	NA	88 (100)	NA	0 (0)	NA

Independent samples t-test was used for continuous variables and Chi-Square test for nominal values. If there were fewer than five units in any of the classes, the Fischer´s exact test was used. Sample size may vary owing to missing values. NA, not applicable, the parameter was excluded by definition from the Control group

Out of the 71 Abusers, 17 (24%) had confirmed use of alcohol, 25 (35%) used alcohol and/or drugs during the ongoing pregnancy, and 29 (41%) were previous risk users fulfilling the inclusion criteria. The mean duration of pregnancy was close to 280 days in all groups (Table 1). Altogether 16.9% of the Abusers gave birth to an SGA infant (Table 2). If alcohol exposure during the ongoing pregnancy was confirmed, an SGA birth was even more prevalent (6 out of 17 (35%)). Placental to birth weight ratio was highest in the Abusers (Table 2).

**Table 2:** Newborn characteristics in each study group

	Controls (*n*=217)	Abusers (*n*=71)	*P*	Smokers (*n*=88)	*P*	SGA mothers (*n*=168)	*P*
Male gender, n (%)	104 (47.9)	44 (62.0)	0.04	52 (59.1)	0.077	90 (53.6)	0.272
Birth weight (g, mean ±SD)	3540±370	3390±560	0.039	3310±550	0.001	2760±400	<0.001
SGA (birth weight <10 percentile), n (%)	0 (0)	12 (16.9)^a^	NA	16 (18.2)	NA	168 (100)	NA
Head circumpherence (cm, mean ±SD)	35.2±1.2	34.9±1.8	0.214	35.0±1.9	0.375	33.8±1.8	<0.001
Head circumpherence (<10th percentile), n (%)	9 (4.1)	14 (19.7)	<0.001	10 (11.4)	0.010	45 (26.8)	<0.001
Intensive care of the newborn, n (%)	0 (0)	15 (21.1)	NA	9 (10.2)	NA	13 (7.7)	NA
Placental weight (g, mean ±SD)	580±106	610±130	0.048	590±131	0.627	460±79	<0.001
Placental weight/birth weight ratio (%, mean ±SD)	16.3±2.5	18.1±2.9	<0.001	17.8±3.3	<0.001	16.8±3.0	0.079

Independent samples t-test was used for continuous variables and Chi-Square test for nominal values. If there were fewer than five units in any of the classes, the Fischer´s exact test was used. Sample size may vary owing to missing values. NA, not applicable, the parameter was excluded by definition from the Control group. ^a^two of these drug abusers

Serum free β-hCG and PAPP-A level comparisons among the Controls and the study groups showed two main findings. Firstly, significantly higher free β-hCG levels were found in the Abusers in comparison to the Controls. Secondly, PAPP-A levels were significantly lower in the SGA mothers and Smokers compared with the Controls. Fetal NTT did not differ significantly between the groups (Table 3).

**Table 3:** Free β-hCG, PAPP-A and NTT in the first trimester screening analysis

	Controls (*n*=217)			Abusers *(n*=71)					Smokers (*n*=88)					SGA mothers (*n*=168)				
	Median	Mean	SD	Median	Mean	SD	MD (95% CI)	*P*	Median	Mean	SD	MD (95% CI)	*P*	Median	Mean	SD	MD (95% CI)	*P*
β-hCG MoM	1.02	1.21	0.72	1.24	1.5	0.88	-0.29 (-0.50, -0.09)	0.013	1.18	1.45	1.06	-0.25 (-0.49, -0.01)	0.048	1.02	1.24	0.82	-0.03 (-0.18, 0.12)	0.700
PAPP-A MoM	1.04	1.20	0.76	0.90	1.14	0.78	0.06 (-0.15, 0.27)	0.197	0.83	0.90	0.47	0.31 (0.16, 0.45)	<0.001	0.90	1.08	0.64	0.12 (-0.02, 0.26)	0.043
NTT MoM	0.90	0.92	0.27	0.89	0.92	0.24	0.01 (-0.06, 0.08)	0.882	0.90	1.02	0.59	-0.09 (-0.19, 0.01)	0.145	0.91	0.98	0.38	-0.06 (-0.12, 0.01)	0.078

Independent samples t-test was used for the free β-hCG, PAPP-A and fetal NTT MoM values in comparison with the Controls. *MD* Mean difference, *CI* 95% confidence interval of the difference, *MoM* Multiples of medians

Stepwise multiple linear regression analyses were performed separately for free β-hCG and PAPP-A levels to explain their variation. Both of these analyses were adjusted for confounding factors (listed in the Methods). Smoking remained the only explaining independent contributor (B = 0.313, beta = 0.15, *P* < 0.001) to high free β-hCG level [F(1,539) = 13.08, *P* < 0.001, R^2^ = 0.024)]. Likewise, smoking (B=-0.21, beta=-0.12, *P* = 0.005) and giving birth to an SGA baby (B=-0.17, beta=-0.12, *P* = 0.007) were the only independent contributors to low PAPP-A levels [F(2,538) = 6.38, *P* < 0.05, R^2^ = 0.023].

Total trisomy risk was higher in the Abusers, Smokers and SGA mothers compared to the Controls (*P* = 0.047, mean difference (MD) of the log10-transformed values − 0.18, 95% CI (-0.35, -0.01), *P* = 0.047; MD -0.28, 95%CI (-0.47, -0.09), *P* = 0.001; MD -0.22, 95%CI (-0.38, -0.06), *P* = 0.007, respectively).

## Discussion

In this study, we got two main findings. Firstly, we found increased free β-hCG levels in the Abusers. Secondly, we found decreased PAPP-A levels in the Smokers and SGA mothers. Fetal NTT did not differ between the groups.

Although our results suggested increased first trimester free β-hCG levels in women using alcohol, there was a significant overlap in free β-hCG levels between the alcohol-exposed and unexposed women. Forty-two % of the Abusers smoked during pregnancy and a multiple regression analysis revealed that smoking explained at least partly the increase in free β-hCG levels in the Abusers. This might be due to a stronger influence of smoking on free β-hCG levels than the correction factor in the FTS protocol predicted. Another possible factor explaining higher free β-hCG levels is that the placental size may have affected hCG production. The Abusers had higher placental weights than the Controls, which could partly explain increased free β-hCG levels. The increase in maternal serum free β-hCG levels in the Abusers could also be a direct effect caused by alcohol exposure, since ethanol treatment of trophoblast cells has been shown to increase hCG production *in vitro* [14, 15]. However, only a small difference and clear overlap between the Abusers and Controls, and the multiple regression analysis results indicated that the association of alcohol abuse with free β-hCG is weak and that free β-hCG cannot be used as a biomarker for alcohol use.

Fetal growth restriction is a classical feature of fetal alcohol syndrome and fetal alcohol effects [16], and the high percentage of SGA births among the Abusers in the present study (17%) is in line with literature [17]. Previous reports on the association between maternal serum free β-hCG levels and fetal growth restriction are conflicting. Fetal growth restriction has been associated with both decreased [18, 19] and elevated free β-hCG [20]. Nevertheless, in the present study, later SGA birth without alcohol or illicit drug exposure did not associate with altred free β-hCG levels.

Our findings of decreased PAPP-A levels in Smokers and SGA mothers are in line with previous studies. Both smoking and fetal growth restriction have previously been associated with decreased PAPP-A values, but the clinical utility of decreased PAPP-A to predict an increased risk to deliver an SGA baby is limited [10, 21–24]. However, regardless of the high prevalence of SGA births and smoking in the Abusers, serum PAPP-A levels of the Abusers were within the low normal range in this study.

The limitations of this study warrant explanation. The retrospective design of this study limits the consistency of the data, especially in the Abusers, where we rely on database information of the abused agents. The relatively small sample size is also a limitation in the study. On the other hand, the AUDIT questionnaire is excellent in identifying dependency, risk drinking, alcohol use disorder or risk drinking [4, 5]. The strengths of the study include the fact that the used serum samples were collected during routine first trimester hospital visits and therefore should represent the variation one would expect to see in clinical samples.

## Conclusions

To our best knowledge, this is the first report on the association between alcohol use during pregnancy and increased FTS free β-hCG levels. Decreased PAPP-A among smokers and SGA mothers was in line with previous studies. The overlap between the study groups was remarkable and we conclude that free β-hCG is not applicable for the use as an alcohol biomarker.

## Abbreviations

free β-hCG: Free β-human chorionic gonadotropin subunit
PAPP-A: Pregnancy-associated plasma protein A
NTT: Nuchal translucency thickness
FTS: First trimester screening
SGA: Small for gestational age
MoM: Multiples of medians
MD: Mean difference

## Availability of data and materials

The data for the current study are not publicly available due to ethical and legal restrictions. However, upon request, data may be available from Anni Lehikoinen at the Department of Pediatrics, Kuopio University Hospital, Finland.
